# Conservative Management of Placenta Percreta: Three Cases and a Review of the Literature regarding Conservative Management of Placenta Accreta Spectrum (PAS) Disorders.

**DOI:** 10.1155/2020/9065342

**Published:** 2020-03-26

**Authors:** M. Patabendige, J. M. P. Sanjeewa, A. M. A. K. G. Amarasekara, R. P. Herath

**Affiliations:** ^1^University Unit of Obstetrics and Gynecology, North Colombo Teaching Hospital, Ragama, Sri Lanka; ^2^Department of Obstetrics and Gynecology, Faculty of Medicine, University of Kelaniya, Ragama, Sri Lanka

## Abstract

**Background:**

The incidence of placenta accreta spectrum (PAS) disorders has risen over the last decades, and there has been a gradual shift towards expectant management. Conservative management of PAS is known to reduce major obstetric haemorrhage and salvage hysterectomy. There is a lack of consensus on the follow-up of patients undergoing conservative approaches. Here, we describe the follow-up of three patients with placenta percreta who were conservatively managed and review the literature for the conservative management of PAS. *Case Presentation*. We have successfully managed three cases of placenta percreta expectantly using combined methods involving symphysial-fundal height, serum beta-HCG, and ultrasonographic volume of placental mass.

**Conclusions:**

Use of a combined approach with symphysial-fundal height, serum beta-HCG, and ultrasonographic volume of placental mass with colour Doppler may guide the surveillance of these conservatively managed cases. However, at least one magnetic resonance imaging three months postoperatively may predict a further risk of delayed haemorrhage.

## 1. Background

The incidence of placenta accreta, increta, and percreta, collectively called placenta accreta spectrum (PAS) disorders, has been rising dramatically over the last decade worldwide, mainly due to a rising caesarean delivery (CD) rate [1, 2]. Antenatal diagnosis and making no attempt to remove any part of the placenta is associated with reduced levels of haemorrhage and therefore less blood transfusions [3, 4]. Although elective caesarean hysterectomy is the standard practice, the choice of conservative management has emerged into practice [1]. Conservation of the uterus reduces numerous short- and long-term complications including massive blood transfusions, disseminated intravascular coagulopathy (DIC), high morbidity/mortality rates, adjacent pelvic organ damage, and infection, as well as long-term psychological sequelae due to the loss of femininity and fertility [5, 6].

The scope of this article is to describe a model for the follow-up of the conservative management of PAS with the placenta in situ approach, while summarizing the follow-up findings of three PAS cases managed in University Unit of Obstetrics and Gynecology, Ragama, Sri Lanka during 2017 and 2018. Sequential changes in symphysial-fundal height, serum beta-HCG, and ultrasonographic volume of placental mass were used as combined methods for the follow-up of these cases. The placental volume was calculated using a 2-dimensional ultrasound scan by measuring the maximum length and anteroposterior and transverse diameters of the uterus and using the formula for the volume of a prolate ellipsoid.

## 2. Case Presentation

### 2.1. Case 1

**Figure 1:**
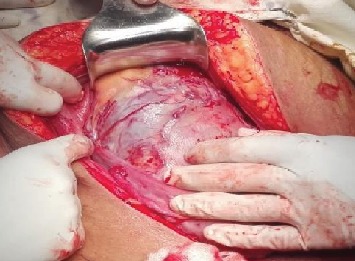
Intraoperative appearance of the uterus and bladder morphologically resembling placenta percreta.

A 33-year-old woman in her third pregnancy, with two previous CD, was found to have an anterior placenta praevia. Two-dimensional (2D) greyscale ultrasonography showed features of morbid adherence (loss of the “clear zone,” presence of abnormal placental lacunae, bladder wall interruption, and myometrial thinning). 2D colour Doppler revealed uterovesical hypervascularity, subplacental hypervascularity, and bridging vessels with possible bladder wall involvement. Her antenatal period was otherwise unremarkable. She underwent a classical CD (without disturbing the placenta) through a midline incision and sterilization at 37 weeks of gestation. The intraoperative appearance of the uterus and bladder morphologically resembled placenta percreta (Figure 1). After delivery of the baby, the cord was ligated at the placental insertion and the uterus was closed keeping the placenta in situ, without any attempt of removing. The intraoperative blood loss was 250 mL. Her recovery was unremarkable. At each follow-up visit, symphysial-fundal height, serum beta-HCG, and ultrasonographic volume of placental mass were measured (Table 1). During the follow-up period, the woman had few episodes of mild vaginal bleeding, which did not need any intervention. Follow-up was done weekly in the first month and then monthly visits until complete resorption of the placenta. She resumed menstruation six months after the delivery.

### 2.2. Case 2

A 35-year-old mother in her third pregnancy, with two previous CD, was found to have an anterior placenta praevia with ultrasound features suggestive of possible bladder wall involvement (like case 1). Her antenatal period was otherwise unremarkable. She underwent a classical CD and sterilization at 36 weeks of gestation. Operative details were similar to case 1. Follow-up details are shown in Table 1. After three months following delivery, the umbilical cord came out into the vagina. The cord was resected at the cervical os, without anaesthesia. There was no attempt to remove the placenta. She was treated with a 5-day course of oral antibiotics. She resumed her first menstruation four months after the delivery.

### 2.3. Case 3

A 29-year-old mother in her third pregnancy, with two previous CD, was diagnosed (ultrasonographic features similar to case 1) to have PAS with possible bladder wall involvement when she presented as having an episode of antepartum haemorrhage at 27 weeks of gestation, which resolved spontaneously. She underwent an elective classical caesarean section and sterilization at 36 weeks of gestation, where placenta percreta with bladder involvement was confirmed. Surgical details and immediate recovery were similar to case 1. Intraoperative blood loss was approximately 300 mL. She was discharged from the ward on postpartum day 10 with a plan of regular follow-up as in previous cases (Table 1). On the 42nd postpartum day, she experienced profuse vaginal bleeding. This was not preceded by fever, abdominal pain, or vaginal discharge. On admission, she was pale and afebrile, had a pulse rate of 120 beats/min, and her blood pressure was 90/50 mmHg. The abdomen was soft and nontender, and the uterus was not palpable abdominally. Vaginal examination revealed continuous bleeding, without passage of placental parts. Her hemoglobin was 6.1 g/dL, and the platelet count was 60 × 10^9^/L. Emergency laparotomy was performed with a midline incision. The uterus was 10 weeks in size, and the ovaries appeared normal. Placental invasion into the bladder from the left side of the uterus was noted. Total abdominal hysterectomy and bilateral internal artery ligation were done by a team composed of an obstetrician, a general surgeon, and a urologist. During the surgery, four units of crossmatched blood, two units of uncrossmatched group-specific blood, two pools of platelets, one pool of fresh frozen plasma, and twelve pools of cryoprecipitate were transfused using guided thromboelastometry, under the supervision of the transfusion medicine specialist. There was no evidence of sepsis to account for DIC. Despite that the second surgery was done six weeks after the primary caesarean section, the surgery was technically demanding due to thick bladder adhesion and associated bleeding. Her postoperative period was uncomplicated. Ultrasound scan of the abdomen along with the kidney, ureters, and bladder was performed and found to be normal.

## 3. Discussion

Traditionally, the treatment of choice for PAS has been hysterectomy due to the risk of massive PPH which is associated with complications such as injuries to the bladder, ureters, and bowel, ovarian damage and infection, massive blood transfusions, disseminated intravascular coagulopathy, and high mortality rates, as well as long-term psychological sequelae due to loss of femininity and fertility [5, 6]. To avoid the disastrous complications of salvage hysterectomy after attempting to remove a morbidly adhered placenta and to preserve fertility, a conservative approach with the placenta in situ was introduced [1, 7, 8].

By leaving a placenta accreta in situ after the delivery of the fetus, one can expect a progressive decrease in blood supply within the uterus, parametrium, and the placenta. This will result in secondary necrosis of the villous tissue, and thus the placenta should progressively detach itself from the uterus (and from the adjacent pelvic organs), finally to resorb or be expelled without significant complications. A large multicenter study in France with 167 cases of PAS disorders found an overall success rate of uterine preservation to be 78% in expectant approach, while the placenta resorbed spontaneously in 75% of cases (median: 13.5 weeks) [9]. However, severe maternal morbidity was seen in 10 (6%) cases in this study. A recent systematic review regarding conservative management of placenta percreta revealed high maternal morbidity (56%) in conservative management [10]. Even amongst conservatively managed cases, complications such as delayed haemorrhage, DIC, endomyometritis, and sepsis uterocutaneous fistula and choriocarcinoma with arteriovenous fistula formation have been reported [11–13]. The aim of the follow-up of these patients is to identify the women at risk for complications for early intervention and to decrease the morbidity rate. We used fundal height, placental volume, and serum beta-HCG in the follow-up.

**Figure 2:**
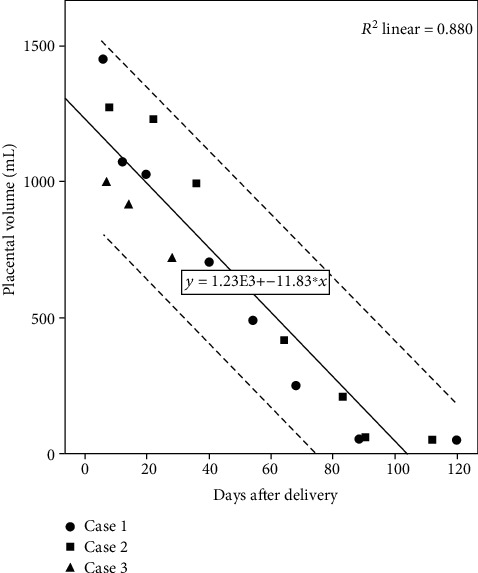
Relationship between regression in the placental volume and days after delivery.

Placental volume measured in ultrasound scan became 50-52 mL after 120 days in case 1 and 112 days in case 2, respectively. The third patient ended up in hysterectomy prior to the above landmarks. Regression in the placental volume seemed to take more than 120 days when we plotted the placental volume of the three cases (Figure 2). Most reviews and guidelines recommended ultrasonographic follow-up for patients undergoing conservative management [14, 15]. Roulot et al. reported in their case series that the appearance of an anechoic range on the remaining placental tissue could announce close elimination of the placenta. Furthermore, they found that the cessation of vascularization of the placental site took on average 47 days, corresponding clinically to the arrest of irregular vaginal bleeding described by the patients [16]. Our third patient, who ended up in secondary haemorrhage, did not reveal an anechoic region before developing secondary haemorrhage.

Measurement of the pulsatility index (PI) of the uterine arteries is a noninvasive method that has been proposed for use in the follow-up of conservative management of placenta accreta [17]. With placental resorption, the low resistance flow of the placental bed disappears, resulting in an increase in PI of uterine arteries. The rising PI is associated with effective conservative management [17, 18].

Methotraxate (MTX) has been used as an adjuvant to expectant management with the aim of expediting placental resorption [19]. MTX, an antifolinic agent acting on rapidly dividing cells, is hypothesized to induce placental necrosis. However, as the placenta does not have rapidly dividing cells, there is a controversy over the use of MTX. Current literature does not show a clear benefit of the use of MTX, adding to the inconclusive evidence of optimal dose and route of administration [1]. Further severe side effects like myelosuppression, nephrotoxicity, and inability to breastfeed, together with inconclusive benefits, precludes the use of MTX [20, 21].

Studies examining pelvic artery embolisation in combination with expectant management have reported success rates of 85–95% [22]. Embolisation can either be done prophylactically during cesarean section or as a treatment to counter haemorrhage. Alanis et al. demonstrated a 76.9% success rate and an 11% complication rate in selective arterial embolization for delayed PPH in placenta increta with conservative management [23]. Bennet and Sen Rahul reported 2 cases of placenta percreta managed conservatively, where bilateral uterine artery embolization was performed prophylactically. They have concluded that the conservative method should only be considered in highly selected cases when blood loss is minimal and there is a desire for fertility preservation [24]. The value of prophylactic placement of balloon catheters in the iliac arteries in cases of PAS disorders is even more controversial, mainly owing to the higher risks of complications than with embolization [1]. Other than expectant management with a placenta in situ approach, three further conservative approaches have been described in the literature, namely, extirpative technique (manual removal of the placenta), one-step conservative surgery (removal of the accreta area), and the Triple-P procedure (suturing around the accreta area after resection), which are beyond the scope of the article [11, 25, 26].

Data from our patients revealed that it took 89 and 90 days for fundus not to become palpable abdominally. Serum beta-HCG level came down to nonsignificant levels only in 68 and 50 days following delivery (Table 1). Duenas-Garcia et al. reported 3 cases where beta-HCG came to <5 IU/L in 21 and 35 days [27]. There is conflicting evidence for serum beta-HCG as a surrogate marker of placental involution [14]. Some studies have suggested that decreasing beta-HCG levels do not correlate with placental involution [19, 27, 28]. Furthermore, undetectable beta-HCG levels do not guarantee complete resorption of retained placental tissue according to previously reported literature [22]. The evidence for this correlation of beta-HCG and placental volume is evolving and newer studies have shown a potential link [29, 30].

Accordingly, an interesting report of five cases of placenta accreta that were conservatively managed showed that placental blood flow disappeared approximately two months after giving birth, almost coincident with a fall in serum beta-HCG [29]. In this study, magnetic resonance imaging (MRI) evaluation of the placenta has supported the usefulness of colour Doppler ultrasonography and serum beta-HCG measurements [29]. However, this same study had a case of delayed postpartum haemorrhage and the authors have emphasized that delayed haemorrhage can occur even at a low but detectable serum beta-HCG level. In our study, we also experienced the same phenomenon in case 3 as described above, but the serum beta-HCG level was approximately 100 IU/mL (Table 1).

Also in our case, the serum beta-HCG levels decreased to minimal levels in three months, just like the volume of the remaining placental tissues in cases 1 and 2 (Table 1). However, it could not predict the possibility of haemorrhage in the third case. Not only that, a Japanese case report on a case of a successful conservative management of the placenta percreta monitored with serial MRI has revealed residual placental tissue even with undetectable serum beta-HCG level around postoperative days 99-103 [30]. This report has also highlighted that an MRI three months postoperatively, may predict the decrease in both blood flow and placental size, hence the risk of delayed haemorrhage [30].

Therefore, it can be concluded that the use of a combined approach with symphysial-fundal height, serum beta-HCG, and ultrasonographic volume of placental mass with colour Doppler may guide the surveillance of these conservatively managed cases. However, at least an MRI three months postoperatively may predict the further risk of delayed haemorrhage. It also needs to be acknowledged that although MRI is informative, it is relatively expensive and not freely available. Since reports suggest that the success rate of conservative management of placenta percreta is lower than that of placenta accreta, careful observation is required for conservative management of placenta percreta [9, 31]. Therefore, the application of MRI in selected high-risk cases can be justified in addition to the aforementioned combined approach.

Conservative management of PAS seems to be a good option even for resource-limited settings, but there must be a strict patient selection policy. The patient's ability for immediate admission to the hospital, their knowledge about the condition, the value of a uterus-preserving option, the willingness for proper follow-up with good compliance, and the availability of a tertiary level emergency surgical access with transfusion facilities in any case of heavy bleeding are essential prior to conservative management. We were successful with both experiences including getting complete resorption and managing a near miss safely.

## 4. Conclusions

Conservative management of placenta percreta appears to be a possible, high-risk alternative to surgical management and should only be reserved for women who refuse standard management of caesarean hysterectomy to preserve fertility or with a strong desire to undergo conservative management. Therefore, strict patient selection is of paramount importance since it may be associated with a higher risk of morbidity and emergency hysterectomy. Sequential changes in symphysial-fundal height, serum beta-HCG, and ultrasonographic volume of placental mass with colour Doppler can be used as combined methods for the follow-up of these cases. A three-month-MRI is a good option to predict the likelihood of delayed haemorrhage.

## 5. Limitations

This paper is not without limitations. Our series included only three cases, and we could not arrive at detailed conclusions due to the small sample size. Moreover, since all three cases are from a single unit, a potential selection bias needs to be considered when interpreting results. A randomized controlled trial can yield robust results regarding the effectiveness of the conservative management of PAS. This should be considered as a research gap on this topic.

## Figures and Tables

**Table 1:** Sequential changes in the clinical and biochemical parameters in three cases.

Case 1				Case 2				Case 3			
Date	SFH (cm)	Serum beta-HCG (IU/mL)	Placental volume (mL)	Date	SFH (cm)	Serum beta-HCG (IU/mL)	Placental volume (mL)	Date	SFH (cm)	Serum beta-HCG (IU/mL)	Placental volume (mL)
Before delivery	35	—	—	Before delivery	36	—	—	Before delivery	38	—	—
Postop day: 06	25	25671	1450	8	24	4478	1272.6	7	28	4521	998.2
Postop day: 12	24	8990	1073	22	22	416	1230.7	14	27	866	912.1
Postop day: 20	24	1258	1030	36	20	22	993.5	28	20	196	712.7
Postop day: 40	20	210	703	50	18	0.82	632.1	40	18	100	621
Postop day: 54	17	19.5	490	64	18	< 0.1	417	—	—	—	—
Postop day: 68	11	< 1.2	248	83	16	—	209	—	—	—	—
Postop day: 89	Not palpable	Not detectable	56	90	—	—	60	—	—	—	—
Postop day: 120	Not palpable	Not detectable	50	112	—	—	52	—	—	—	—

Postop day: number of days after caesarean delivery; beta-HCG: beta-chain of human chorionic gonadotrophin; SFH: symphysiofundal height; cm: centimeters; mL: milliliters.

## Abbreviations

PAS: Placenta accreta spectrum
Beta-HCG: Beta-human chorionic gonadotropin
2D: Two dimensional
CS: Caesarean section
DIC: Disseminated intravascular coagulation
FIGO: International Federation of Gynecology and Obstetrics
PPH: Postpartum haemorrhage
MTX: Methotrexate.
